# Experimental Investigation into the Failure Mechanisms of Fabric Membrane Materials with Defective Flat Round Cracks

**DOI:** 10.3390/ma17122970

**Published:** 2024-06-17

**Authors:** Wenrui Li, Ping Liu, Jiancheng Zhang, Sakdirat Kaewunruen

**Affiliations:** 1 School of Civil Engineering and Architecture, Jiangsu University of Science and Technology, Zhenjiang 212004, China; lwenrui2022@stu.just.edu.cn (W.L.); liupinghaiyan@just.edu.cn (P.L.); zjc0508@just.edu.cn (J.Z.); 2 Department of Civil Engineering, School of Engineering, University of Birmingham, Birmingham B15 2TT, UK

**Keywords:** oblate initial crack, membrane material, crack propagation, failure mechanism

## Abstract

The emphasis of this study is placed on the investigation into the failure mechanisms of the fabric membranes when exposed to such defective cracks. This experimental study investigates the initial crack of a flat circle and conducts a uniaxial shear test on the membrane materials. The deformation of the membrane materials is obtained through an optical non-contact scanner. Our study has been conducted to assess the crack propagation of fabric membrane materials at different angles. The relationships between crack width and stress together with stress and strain are also obtained. Based on the results, a mechanic of failure on the membrane was proposed. Moreover, new findings into the ductility and energy absorption of the fabric membrane materials have been established to inform the failure mechanisms.

## 1. Introduction

**Figure 1:**
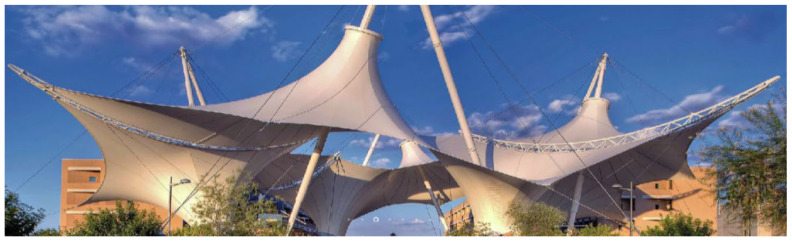
The mast-supported canopy in the city of Scottsdale.

Structural membrane materials with their lightweight, high-strength properties, are widely used in various fields such as public buildings, emergency rescue, and so on, and can afford excellent architectural expressions and large application spaces [1,2,3]. Membrane structures have widespread applications, which usually use the PVDF membrane. PVDF membranes are made of yarns and coating. The coating is high-quality polyvinylidene fluoride (PVDF) and provides high performance for buildings. The yarns are usually polyester or fiberglass, which offers the strength of mechanics. The combination of yarns and coating can provide good mechanical strength and excellent chemical properties including being waterproof, thermal insulation, corrosion-resistant, etc. Numerous instances of damage and even accidents leading to destruction have emerged [4,5]. The failure of membrane materials generally does not result from the ultimate tensile strength obtained when the material is undamaged [6,7]. Instead, it stems from damages such as broken filaments, cracks, and creases occurring during the production, processing, and construction processes [8,9]. Stress concentration near these defects can lead to tearing failure at low stress levels, making tearing the primary failure mode of membrane structures. Consequently, understanding the durability and tearing mechanisms of membrane structures has become increasingly important [10,11,12,13]. The tensile membrane structure is shown in Figure 1 [14].

This research topic has been researched by scholars both domestically and internationally. Chen et al. [15] conducted experimental research on the tear performance of biaxial warp-knitted fabrics with different off-axis angles. The results showed that in off-axis tensile tests, the lowest tensile strength of biaxial warp-knitted fabrics occurred at the off-axis angles of 15° and 75°. Zhang et al. [16] studied the fitting effect of different treatment methods on the nonlinear uniaxial tensile properties of two coated fabrics based on experimental results and obtained reasonable fracture toughness and accurately predicted critical tear strength. T. Ennouri et al. [17] conducted a tensile center crack tearing test, developed a textile structure standard based on tearing energy by using composite textile materials, and studied the effects of fabric linear density, yarn density, and weaving on tearing behavior. M. Pankow et al. [18] studied tear tests on two different weaving structures to determine how failure begins in composite materials in each case. The results showed that there exists a strain field dependent on the structure of these materials, and this localization led to the onset of failure.

At present, the research on damaged membrane materials at home and abroad mainly focuses on the tearing strength and tearing process of the membrane materials, and the results are all based on the assumption that the membrane materials are isotropic [19,20,21,22]. In practice, however, the film is typically a discrete material, its strength is determined by the baseline, and the fracture of the material is determined by the yarn. In field tests, only the lateral deformation of the crack plane can be observed in the specimens, and there are few representations of the deformation of the tested membrane material in three dimensions. Therefore, this study primarily focuses on analyzing the deformation stages of tension membranes with oblateness initial cracks in uniaxial shear tests. Using an optical scanner, the study investigates changes in the stress state of the membrane material and the deformation morphology of the tension membrane at the moment of final tearing, establishing a relationship between crack width and stress function.

## 2. Experiments

### 2.1. Test Settings

The experiment has conducted uniaxial shear testing on membrane materials using a new innovative shear apparatus for soft membrane materials developed at Jiangsu University of Science and Technology. The dimensions of the membrane material are 200 mm × 50 mm, and its relevant properties are listed in Table 1. The material in the test is PVDF, which was usually used in the application with its high strength, chemical resistance, thermal stability, etc. The edges of the membrane material have been curled at both ends for easy clamping by fixtures. Considering the material’s anisotropy, the study investigated the influence of initial cut deflection angle on yarn orientation. Test data are then analyzed to obtain relevant data on membrane crack and overall deformation diagram.

**Figure 2:**
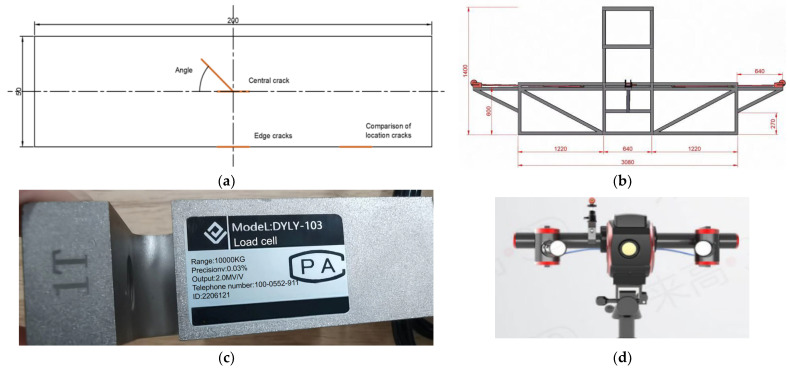
Test specimens and devices. (**a**) Initial crack location of the specimen. (**b**) Device dimension diagram. (**c**) DYLY-103 S sensors for tension force. (**d**) Q7 optical scanner.

The initial crack position of the specimen is shown in Figure 2a, and the experimental setup is depicted in Figure 2b. The deformation of the specimen under each load can be captured using an optical scanner, which is shown in Figure 2d, and the load can be measured using an S-type load cell shown in Figure 2c. In the experiments, the load of 500 N has been added step by step in each stage.

### 2.2. Test Conditions

To investigate the impact of crack position and angle on crack propagation and failure mechanism, oval-shaped cracks are placed at the center and edges of the membrane materials, with angles selected at 0°, 30°, 45°, 75°, and 90° for five different conditions. During the experiments, the loads for the 0°, 30°, 45°, 75°, and 90° conditions are set at 500 N in stages, as outlined in Table 2. In Table 2, “***a***” represents the major axis of the flat circle, and “***b***” represents the minor axis of the flat circle.

### 2.3. Data Processing Methods

The circular holes in the on-site image and the point cloud image are both identification points for the optical scanner, with no data collection involved. They are solely used for acquiring and identifying the point cloud. Stress refers to the average stress on the membrane material, strain represents the ratio of the length increment due to deformation in the corresponding condition direction to the original length, crack length is the maximum length between the crack tips in the corresponding condition direction, crack width is the maximum length of the crack in the perpendicular direction to the corresponding condition, and crack vertical displacement is the maximum displacement between the original plane of the membrane material and the vertical deformation point of the crack under the corresponding load.

## 3. Experimental Results

### 3.1. Stress and Strain Results

**Figure 3:**
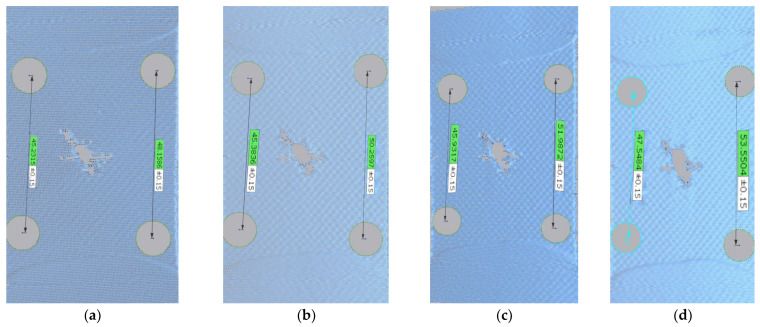
Point cloud diagram of membrane material under the case of 75°: (**a**) 0 N; (**b**) 500 N; (**c**) 1000 N; and (**d**) 1500 N.

The point cloud diagram of 75° is shown in Figure 3. Based on the point cloud in Figure 3, the stress and strain can be measured. It can be seen clearly that the initial crack is relatively small, and it later extends with the increase in the load. It roughly seems like an elliptical shape at the load of 1500 N. Although the shape of the crack has not changed significantly, its elongation can be seen from the data in Figure 3.

#### 3.1.1. Force–Displacement

**Figure 4:**
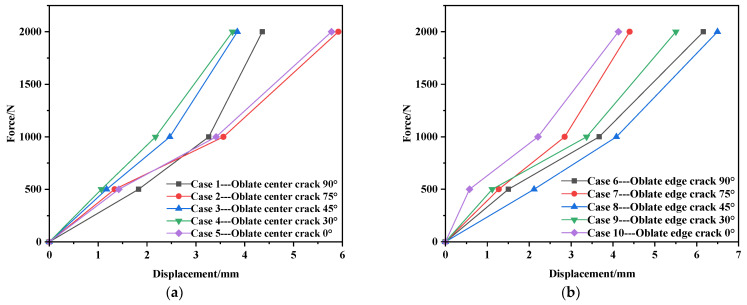
Force–displacement graphs. (**a**) Case of center. (**b**) Case of edge.

Force–displacement graphs are shown in Figure 4. It can be seen from Figure 4 that the specimen is in the stage of elastic deformation during the tensile process.

#### 3.1.2. Cases of Central Crack

**Figure 5:**
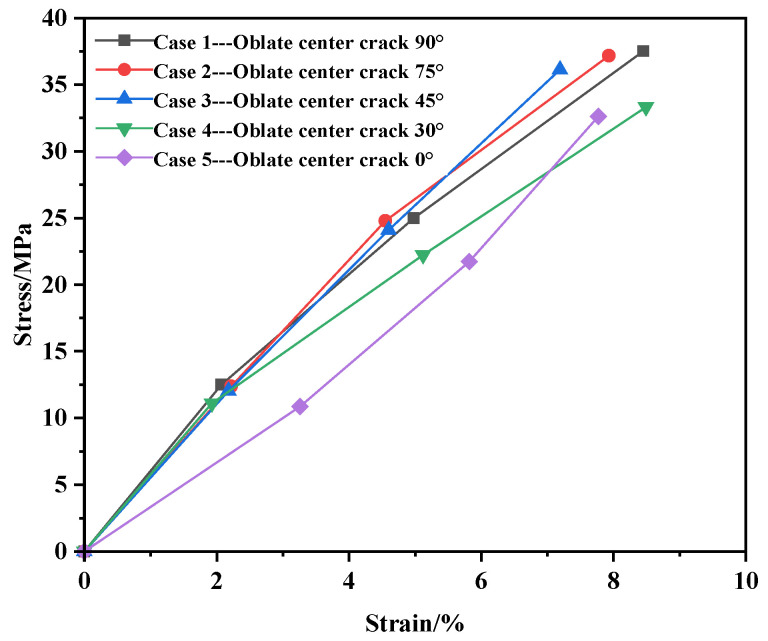
Stress–strain diagram at the center position.

Under the central crack cases, the stress–strain values of the membrane material are shown in Table 3, and the stress–strain curve is shown in Figure 5.

As shown in Figure 5, the stress–strain curves for all five conditions exhibit a linear relationship. The stress–strain curve formula for the central position has been fitted using MATLAB, yielding Equation (1) as shown below, where σ represents stress and ε represents strain, ***E*** can be the elastic module, which is approximately 440 MPa.

(1) σ=E⋅ε

#### 3.1.3. Cases of Edge Crack

**Figure 6:**
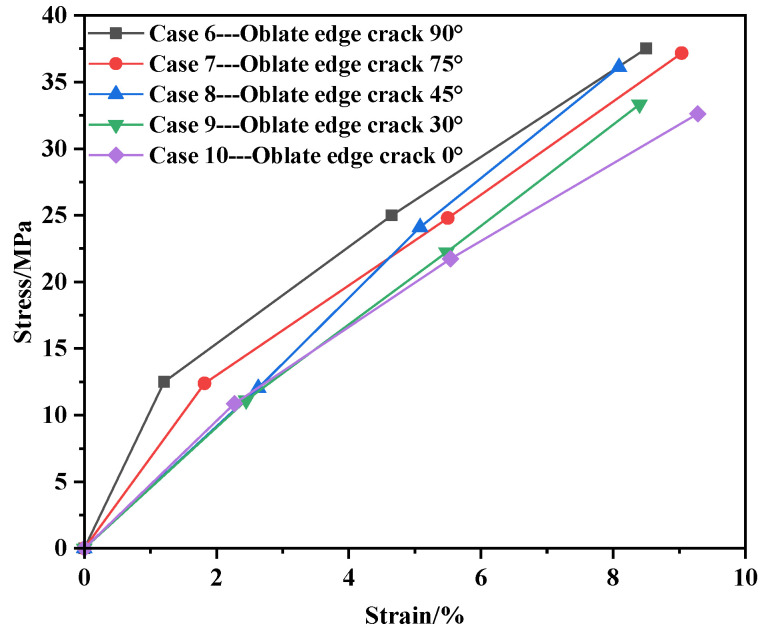
Stress–strain diagram at the edge position.

Under the condition of edge cracks, the stress–strain values of the membrane material are shown in Table 4, and the stress–strain curve is shown in Figure 6.

Consistent with the central initial crack, Figure 6 reveals that the stress–strain curves for all five conditions exhibit a linear relationship. By fitting the stress–strain curve formula for the edge position using MATLAB, it aligns with Equation (1), indicating that Equation (1) corresponds to the stress–strain trend of membrane materials with edge-shaped cracks under uniaxial tension.

Based on the data presented above, it can be concluded that during the initial stage of external tensile stress, when the stress is relatively low, the strain distribution is relatively uniform, and the deformation of the specimen is generally consistent throughout. As the external tensile stress increases, the strain distribution of the specimen becomes uneven, with larger strain regions forming at the ends of the crack. Moreover, the strain increases closer to the crack ends. This is primarily because the presence of the initial crack causes a significantly greater deformation of yarns at the crack ends compared to those further away from the crack, leading to an uneven internal yarn deformation that results in stress concentration at the crack ends.

### 3.2. Crack Propagation

**Figure 7:**
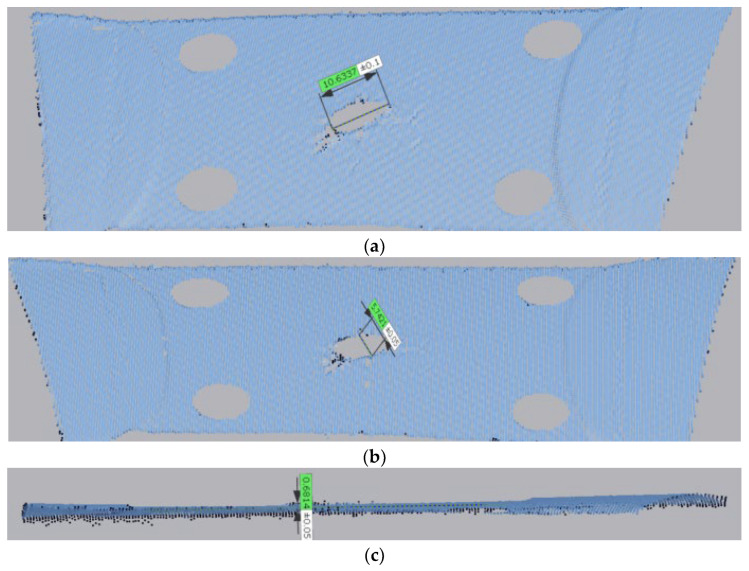
The method to measure the parameter of crack. (**a**) The length of the crack. (**b**) The width of the crack. (**c**) The vertical displacement of the crack.

Figure 7 displays the method to measure the parameters of the crack such as the length, width, and vertical displacement of the crack.

#### 3.2.1. Central Crack Case

**Figure 8:**
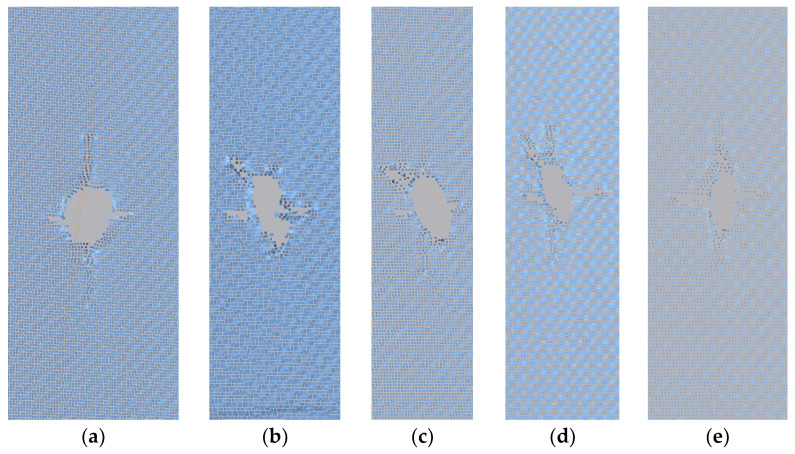
The final form before failure at the center position: (**a**) 90°; (**b**) 75°; (**c**) 45°; (**d**) 30°; and (**e**) 0°.

**Figure 9:**
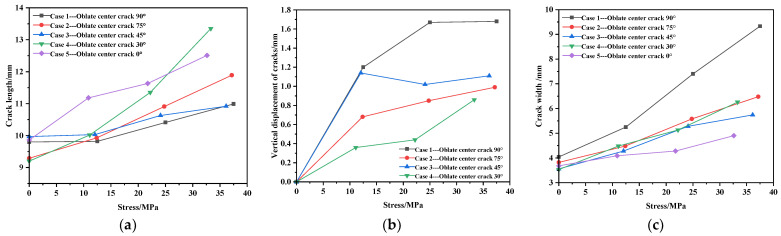
Central crack propagation-related curve. (**a**) Stress–length curve. (**b**) Stress–vertical displacement curve. (**c**) Stress–width curve.

The final point cloud shape of each working condition at the center position is shown in Figure 8. The length, width, and vertical displacement values of cracks are shown in Table 5. Central crack propagation-related curves are shown in Figure 9.

From Figure 8, it can be observed that the deformation at 90° is the most obvious. The crack propagation changes from oval to circular.

From Table 5 and Figure 9, it can be observed that as the external load increases during the deformation process, the area of the defect also increases. During stretching, the initial oblateness crack evolves into a circular shape, with the crack propagation direction always following the preset direction. The crack gradually widens and exhibits an increase in vertical displacement. After reaching the midpoint of the external load, the vertical deformation maintains minor fluctuations. The crack length generally shows a linear growth trend. Under the 90° condition, the crack in the membrane material expands at the fastest rate, reaching a circular shape at 1500 N, with the crack width approaching its length.

Table 5 and Figure 9 show that under the 0° condition, the crack length expansion is more pronounced compared to the width in the membrane material.

**Figure 10:**
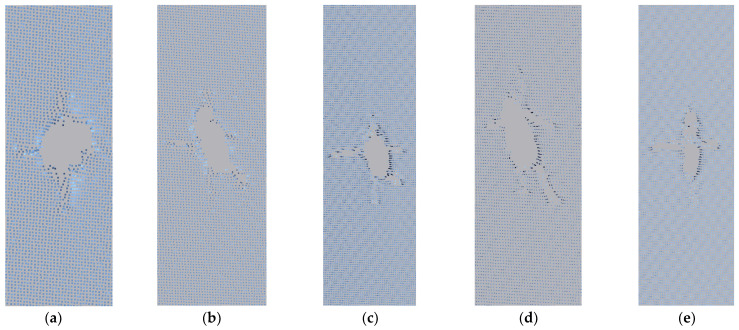
The final form of each case at the edge position: (**a**) 90°; (**b**) 75°; (**c**) 45°; (**d**) 30°; and (**e**) 0°.

From Figure 10a, it can be observed that the crack length is positively correlated with stress across all five conditions. Figure 10b shows that initially, there is a linear relationship between stress and vertical crack displacement, which later stabilizes gradually. The crack width is directly proportional to stress, forming a function relationship. The stress–width curve for the central position, calculated using MATLAB, is shown in Figure 10c and is represented by Equation (2), where w represents crack width, σ represents stress value, and $w_{0}$ represents the initial cut width. Theoretically, the initial crack width should be the minor axis of the oval shape, which in this experiment is approximately 4.0 mm.

(2) $$w=0.067\cdot \sigma +w_{0}$$

#### 3.2.2. Edge Crack Working Condition

The final shape of each case at the edge position is shown in Figure 10.

**Figure 11:**
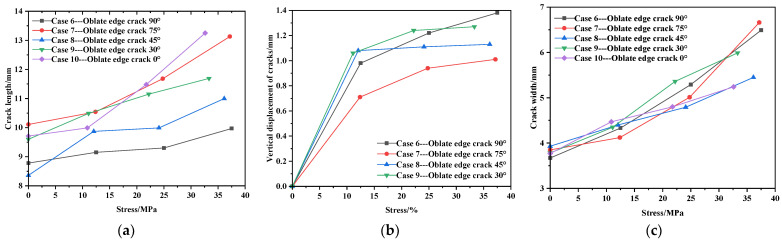
Edge crack propagation-related curve. (**a**) Stress–length curve. (**b**) Stress–vertical displacement curve. (**c**) Stress–width curve.

Consistent with the central crack, the vertical displacement of the crack under the 0° working condition is not significant and cannot be measured, so these data are not included in this working condition. The length, width, and vertical displacement values of cracks are shown in Table 6, and the relevant curve graphs are shown in Figure 11.

From Table 6 and Figure 10, it can be deduced that under the 90° case, the crack in the membrane material expands at the fastest rate, while under the 0° condition, crack deformation is less pronounced. Compared to the central initial crack, edge cracks exhibit a more significant expansion in crack width during the propagation process. From Figure 10, the deep blue area on the edge of the crack is buckling. It can be inferred that during stretching, the membrane material exhibits curling at the edges. This is because the applied force causes transverse contraction in the membrane material, leading to curling to release stress. The membrane material curls towards the side with lower stress. The crack length shows a relatively gradual linear growth trend.

Consistent with the central initial crack, Figure 11a reveals that the crack length is positively correlated with stress across all five conditions. Figure 11b indicates that there is initially a linear relationship between stress and vertical crack displacement, which later stabilizes gradually. The crack width is directly proportional to stress, forming a function relationship. The stress–width curve for the edge position, calculated using MATLAB, is shown in Figure 11c, and the fitted function is consistent with Equation (2).

Figure 11c illustrates that at low stress levels, the crack width increases rapidly initially but then increases very minimally. This is because the initial cut may not be at the baseline position, and the mechanical properties of the coating are poor, making it prone to tearing. Once the crack expands to the baseline, the crack width essentially stops increasing.

## 4. Discussion

### 4.1. Tip Stress

According to fracture mechanics theory, the tip stress $\sigma_{\max}$ at the edge stress is represented by Equation (3) under the assumption of the cut being elliptical [23]. Where $\sigma_{aver}$ is the average stress on the hole ligament, 2a is the vertical length of the cut, 2b is the horizontal length of the cut, and 2h is the width of the membrane material.

(3) $$\begin{array}{c} \sigma_{\max}=S^{\prime }\sigma_{aver}\\ S^{\prime }=\{\left(1+2a/b\right){\left(a-b\right)}^{2}\left(1-a/h\right)h\}/\{\left(b^{2}/2-ab\right)h+\\ \left(a^{2}-ab+b^{2}/2\right)\sqrt{h^{2}+b^{2}-a^{2}}+\left[\left(a-b\right)b^{3}/2\right]/\sqrt{h^{2}+b^{2}-a^{2}}\} \end{array}$$

By using Equation (3) for calculations, the tip stress under each condition can be obtained, as shown in Table 7. From the tip stress values, it can be observed that among the 10 conditions, only the 75° condition has a tip stress exceeding 120 MPa, but none of them reach the ultimate stress of the membrane material yarns. Therefore, the membrane material does not exhibit complete fracture.

### 4.2. The Mechanic of Failure

**Figure 12:**
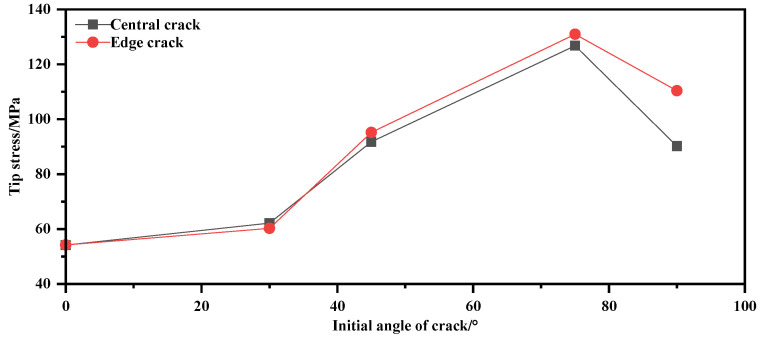
Tip stress–angle relationship diagram.

**Figure 13:**
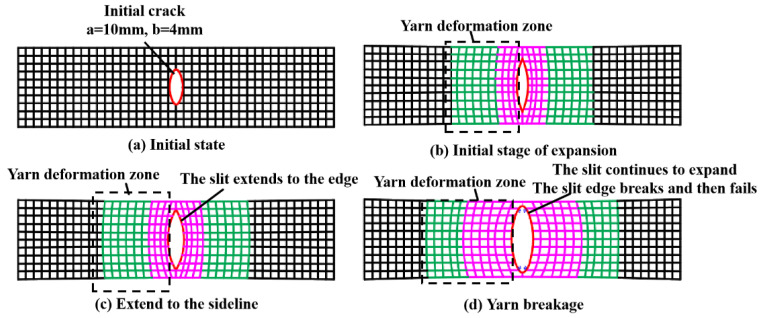
The mechanic of the process from crack to failure.

Tip stress under various cases is shown in Table 7. The tip stress–angle relationship diagram is shown in Figure 12. The mechanics of the process from crack to failure are shown in Figure 13.

From Figure 12 and Table 7, it can be seen that when the angle is 0°, the tip stress is very small. However, as the angle exceeds 30°, the tip stress starts to increase, reaching its maximum at 75°. This is because, at small angles, the membrane material can be considered isotropic, resulting in minimal variation in tip stress. On the other hand, at larger angles, the tip stress approaches the baseline strength, and a single baseline approaches the tearing state while extending to other baselines, causing the tip stress to increase rapidly.

Based on the above, Figure 13 is obtained. Figure 13 depicts the deformation of the film yarn during stretching. The red area and the green area are obvious areas of yarn deformation. Among them, the deformation of the red region is greater than that of the green region. The deformation of the remaining black areas is not obvious.

### 4.3. Energy Absorption

**Figure 14:**
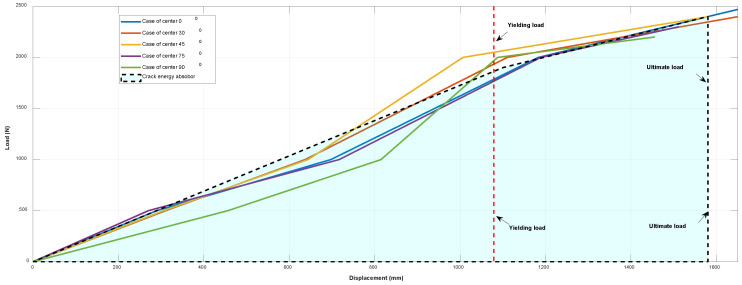
The diagram of load vs. displacement on center cases.

Figure 14 portrays the load behaviors of membrane materials against displacements on the center cases. It should be noted that the ultimate load or maximum load construes the membrane failure. Our experimental results demonstrate the elasticity of membrane materials before yielding. From the load–displacement behaviors, the energy absorption of failure can be obtained (from the area under the load–deflection curve) as illustrated in Figure 14.

From the point view of energy absorption, it can be seen that the ratio of the energy at failure over the energy of elastic is,

(4) $$E_{u}/E_{e}\approx 1.98$$

Which shows that the material can consume about the same energy of elastic after yield load. In addition, the ductility ratio of the membrane materials can be calculated as $\sigma_{y}/\sigma_{e}$. In this present study, the ductility ratio is about 1.44, which is lower than the energy ratio. This is because the plastic stage can absorb more energy.

## 5. Conclusions

This study focuses on the deformation analysis of membrane materials in various failure states based on uniaxial shear tests. Using optical scanning technology, three-dimensional deformation data of membrane materials with initial cracks under tension are obtained. Subsequently, the expansion and evolution mechanisms of defects (cracks) in the membrane materials have been analyzed, and it can enhance the design of the membrane structure. Our experimental study reveals the new findings as follows:

1. The crack width increases continuously during the stretching process, initially increasing rapidly. As stress increases, the crack width increment becomes small, indicating a transition to a plastic state. The crack width is directly proportional to stress, forming a function relationship. Equation (2) can be used to approximate the calculation of crack width.
2. Simultaneously, during the evolution of crack width, the membrane material may undergo curling, but this curling does not affect the mechanical properties of the membrane material.
3. The length of the crack increases with the increase in load, suggesting that the crack point may not always be at a fiber position but could also be at a baseline position. Once the crack point reaches a fiber position, its length no longer increases.
4. As stress increases, the strain distribution of the membrane material becomes uneven, with greater strain near the crack ends. Inconsistent internal yarn deformations lead to stress concentration at the crack ends. Stress is directly proportional to strain, forming a function relationship. Equation (1) can be used to approximate the calculation of crack strain.

## Figures and Tables

**Table 1:** Performance parameters of membrane materials.

Parameter	Type	Base Density	Elastic Module	Tension Strength	Tear Strength	Yarn Thickness	Yarn Strength
Data	PVDF	1300 g/m^2^	430~480 MPa	9000 N/5 cm	550 N/5 cm	1500 D × 1500 D	~100 N

**Table 2:** Test conditions.

Case	Position	Crack Shape	Crack Angle	Initial Crack Length
1	Center	Oblate circle	90°	***a*** = 10 mm, ***b*** = 4 mm
2	Center	Oblate circle	75°	***a*** = 10 mm, ***b*** = 4 mm
3	Center	Oblate circle	45°	***a*** = 10 mm, ***b*** = 4 mm
4	Center	Oblate circle	30°	***a*** = 10 mm, ***b*** = 4 mm
5	Center	Oblate circle	0°	***a*** = 10 mm, ***b*** = 4 mm
6	Margin	Oblate circle	90°	***a*** = 10 mm, ***b*** = 4 mm
7	Margin	Oblate circle	75°	***a*** = 10 mm, ***b*** = 4 mm
8	Margin	Oblate circle	45°	***a*** = 10 mm, ***b*** = 4 mm
9	Margin	Oblate circle	30°	***a*** = 10 mm, ***b*** = 4 mm
10	Margin	Oblate circle	0°	***a*** = 10 mm, ***b*** = 4 mm

**Table 3:** Stress–strain on the cases of central crack.

Parameter	Stress/MPa					Strain/%				
Load/N	1 −90°	2 −75°	3 −45°	4 −30°	5 −0°	1 −90°	2 −75°	3 −45°	4 −30°	5 −0°
0	0	0	0	0	0	0	0	0	0	0
500	12.50	12.39	12.05	11.11	10.87	2.07	2.22	2.18	1.93	3.26
1000	25.00	24.79	24.10	22.22	21.74	4.98	4.55	4.60	5.12	5.82
1500	37.50	37.18	36.15	33.33	32.61	8.45	7.93	7.19	8.49	7.77

**Table 4:** Stress–strain under the cases of edge crack.

Parameter	Stress/MPa					Strain/%				
Load/N	6 −90°	7 −75°	8 −45°	9 −30°	10 −0°	6 −90°	7 −75°	8 −45°	9 −30°	10 −0°
0	0	0	0	0	0	0	0	0	0	0
500	12.50	12.39	12.05	11.11	10.87	1.21	1.82	2.63	2.45	2.27
1000	25.00	24.79	24.10	22.22	21.74	4.65	5.50	5.08	5.48	5.54
1500	37.50	37.18	36.15	33.33	32.61	8.50	9.04	8.09	8.40	9.28

**Table 5:** Numerical values related to central crack propagation.

**Parameter**	**Stress/MPa**					**Crack Length/mm**				
Load/N	1 −90°	2 −75°	3 −45°	4 −30°	5 −0°	1 −90°	2 −75°	3 −45°	4 −30°	5 −0°
0	0	0	0	0	0	9.80	9.29	9.97	9.20	9.86
500	12.50	12.39	12.05	11.11	10.87	9.82	9.93	10.03	10.02	11.18
1000	25.00	24.79	24.10	22.22	21.74	10.41	10.91	10.63	11.35	11.63
1500	37.50	37.18	36.15	33.33	32.61	10.99	11.89	10.92	13.35	12.51
**Parameter**	**Crack Width/mm**					**Vertical Displacement of Cracks/mm**				
Load/N	1 −90°	2 −75°	3 −45°	4 −30°	5 −0°	1 −90°	2 −75°	3 −45°	4 −30°	5 −0°
0	4.04	3.82	3.56	3.53	3.68	/	/	/	/	/
500	5.25	4.48	4.28	4.48	4.09	1.20	0.68	1.14	0.36	
1000	7.40	5.59	5.28	5.13	4.28	1.67	0.85	1.02	0.45	
1500	9.33	6.48	5.74	6.26	4.90	1.68	0.99	1.11	0.86	

**Table 6:** Numerical values related to edge crack propagation.

**Parameter**	**Stress/MPa**					**Crack Length/mm**				
Load/N	6 −90°	7 −75°	8 −45°	9 −30°	10 −0°	6 −90°	7 −75°	8 −45°	9 −30°	10 −0°
0	0	0	0	0	0	8.78	10.11	8.36	9.60	9.72
500	12.50	12.39	12.05	11.11	10.87	9.15	10.54	9.87	10.49	9.99
1000	25.00	24.79	24.10	22.22	21.74	9.30	11.68	9.99	11.15	11.48
1500	37.50	37.18	36.15	33.33	32.61	9.97	13.13	11.00	11.69	13.25
**Parameter**	**Crack Width/mm**					**Vertical Displacement of Cracks/mm**				
Load/N	6 −90°	7 −75°	8 −45°	9 −30°	10 −0°	6 −90°	7 −75°	8 −45°	9 −30°	10 −0°
0	3.67	3.85	3.93	3.78	3.77	/	/	/	/	/
500	4.33	4.12	4.40	4.35	4.47	0.98	0.71	1.08	1.06	
1000	5.29	5.01	4.79	5.36	4.80	1.22	0.94	1.11	1.24	
1500	6.49	6.66	5.45	5.99	5.24	1.38	1.01	1.13	1.28	

**Table 7:** Tip stress under various cases.

Parameter	Central Crack Condition/MPa					Edge Crack Working Condition/MPa				
Case Angle	1–90°	2–75°	3–45°	4–30°	5–0°	6–90°	7–75°	8–45°	9–30°	10–0°
0	0	0	0	0	0	0	0	0	0	0
500	42.77	50.44	35.44	21.65	17.71	47.70	55.90	34.49	22.42	19.18
1000	68.54	89.98	63.61	42.69	35.45	82.40	101.69	65.61	41.49	37.13
1500	90.18	126.76	91.74	62.16	54.16	110.37	130.95	95.17	60.24	54.21

## Data Availability

The data that support the findings of this study are available from the corresponding author upon reasonable request.

## Abbreviations

PVDF	polyvinylidene fluoride
***a***	the major axis of the flat circle
***b***	the minor axis of the flat circle
σ	represents stress
ε	represents strain
***E***	can be the elastic module
$\sigma_{\max}$	under the assumption that it is cut as an ellipse, the edge stress is represented and is expressed in Equation (3)
$\sigma_{aver}$	average stress on the hole ligament
2a	the vertical length of the cut
2b	the horizontal length of the cut
2h	the width of the membrane material
$E_{u}$	the energy at failure
$E_{e}$	the energy of elastic
